# Supplementary material for: Behavioral flexibility in an invasive bird is independent of other behaviors
Source: PeerJ. 2016 Jul 12;4:e2215. doi: 10.7717/peerj.2215 (PMC4950539; doi:10.7717/peerj.2215)
Supplement: Supplemental Information 1 — GLM outputs (Tables S1 and S2). [file peerj-04-2215-s001.docx]

***Supplementary Material for:***

Logan CJ. 2016. Behavioral flexibility in an invasive bird is independent of other behaviors. *PeerJ* 4:e2215. doi:10.7717/peerj.2215. <https://peerj.com/articles/2215/>

**Table S1. Does behavioral flexibility correlate with exploration or persistence?** GLM results with behavioral flexibility scores (response variable), exploration (total number of section changes) and persistence (total number of interactions with the table, apparatus, and stick) while examining the potential influence of batch (explanatory variables). Poisson distribution with a log link, n=7 birds, CI=Bayesian credible interval.

|  | **Posterior mean** | **95% CI** | **p** |
| --- | --- | --- | --- |
| Intercept | 4.03 | -1.60-9.54 | 0.11 |
| Exploration | 0.004 | -0.04-0.05 | 0.87 |
| Persistence | 0.00006 | -0.007-0.007 | 0.93 |
| Batch | -0.08 | -7.25-6.21 | 0.93 |

**Table S2. Does behavioral flexibility correlate with risk aversion or motor diversity?** GLM results with behavioral flexibility scores (response variable), risk aversion (percent time spent in the safest sections of the aviary) and motor diversity (total number of motor actions used) while examining the potential influence of batch (explanatory variables). Poisson distribution with a log link, n=7 birds, CI=Bayesian credible interval.

|  | **Posterior mean** | **95% CI** | **p** |
| --- | --- | --- | --- |
| Intercept | 8.62 | -6.85-25.93 | 0.20 |
| Risk aversion | -0.05 | -0.21-0.11 | 0.41 |
| Motor diversity | 0.06 | -0.22-0.35 | 0.51 |
| Batch | -0.28 | -2.65-2.00 | 0.75 |
